# Internet Versus Mailed Questionnaires: A Randomized Comparison (2)

**DOI:** 10.2196/jmir.6.3.e30

**Published:** 2004-09-24

**Authors:** Pam Leece, Mohit Bhandari, Sheila Sprague, Marc F Swiontkowski, Emil H Schemitsch, Paul Tornetta, PJ Devereaux, Gordon H Guyatt

**Affiliations:** ^6^Department of MedicineMcMaster UniversityHamilton ONCanada; ^5^Department of Orthopaedic SurgeryBoston UniversityBoston MAUSA; ^4^Department of Orthopaedic SurgerySt. Michael's HospitalToronto ONCanada; ^3^Department of Orthopaedic SurgeryUniversity of MinnesotaMinneapolis MNUSA; ^2^Department of Orthopaedic SurgeryDepartment of Clinical Epidemiology and BiostatisticsMcMaster UniversityHamilton ONCanada; ^1^Department of Clinical Epidemiology and BiostatisticsMcMaster UniversityHamilton ONCanada

**Keywords:** Survey, methods, mail surveys, Internet surveys, response rate

## Abstract

**Background:**

Low response rates among surgeons can threaten the validity of surveys. Internet technologies may reduce the time, effort, and financial resources needed to conduct surveys.

**Objective:**

We investigated whether using Web-based technology could increase the response rates to an international survey.

**Methods:**

We solicited opinions from the 442 surgeon–members of the Orthopaedic Trauma Association regarding the treatment of femoral neck fractures. We developed a self-administered questionnaire after conducting a literature review, focus groups, and key informant interviews, for which we used sampling to redundancy techniques. We administered an Internet version of the questionnaire on a Web site, as well as a paper version, which looked similar to the Internet version and which had identical content. Only those in our sample could access the Web site. We alternately assigned the participants to receive the survey by mail (n=221) or an email invitation to participate on the Internet (n=221). Non-respondents in the mail arm received up to three additional copies of the survey, while non-respondents in the Internet arm received up to three additional requests, including a final mailed copy. All participants in the Internet arm had an opportunity to request an emailed Portable Document Format (PDF) version.

**Results:**

The Internet arm demonstrated a lower response rate (99/221, 45%) than the mail questionnaire arm (129/221, 58%) (absolute difference 13%, 95% confidence interval 4%-22%, P<0.01).

**Conclusions:**

Our Internet-based survey to surgeons resulted in a significantly lower response rate than a traditional mailed survey. Researchers should not assume that the widespread availability and potential ease of Internet-based surveys will translate into higher response rates.

## Introduction

Health-care surveys are an important research tool to study the attitudes, beliefs, behaviors, practice patterns, and concerns of physicians [1]. Response rates to surveys, especially among physicians, have been suboptimal (mean response rates=62%, SD=15%) [2]. Investigators have attributed the lower response rates to increasing physician workloads and to the low priority physicians place on survey completion. The return rates have been especially low in surveys of surgeons, who have responded at rates from 15%-77% [3-6]. Low response rates threaten the validity of a survey by increasing the risk of a non-response bias [1,7,8].

Dillman's Tailored Design Method is the current standard for conducting mail and Internet surveys [9]. A recent Cochrane Methodology Review verified the success of these strategies for achieving reproducible response rates in the general population [10,11]. Another systematic review also confirmed that some of these methods are effective in physician surveys: monetary incentives, stamps on outgoing and return envelopes, and short questionnaires [1].

The suboptimal response rates among surgeons calls for exploration of alternative survey administration strategies. Internet technology has the potential to decrease the time and cost involved in conducting a health-care survey. Couper presents a review of issues and approaches to Web surveys, and suggests that Web surveys may improve the response rate and lower the cost of surveys [12]. While some Internet-based surveys have shown promising response rates (up to 94% [13]), their potential has not been realized in other studies (response rates ranged from 11%-70%) [14-17]. To date, no studies have evaluated the response rates to Internet surveys among orthopaedic surgeons.

We hypothesized that orthopaedic surgeons who were given the opportunity to participate in an Internet-based questionnaire would respond at a higher rate than surgeons who were mailed a paper copy of the survey. We tested this hypothesis in a survey of orthopaedic surgeons on their views about managing hip fractures.

## Methods

### Questionnaire Development

We developed an 8-page self-administered questionnaire to identify the preferences and practice patterns of orthopaedic traumatologists in the operative treatment for femoral neck fractures. Using previous literature, focus groups with orthopaedic surgeons, and key informants, using sampling to redundancy techniques, we identified items that fell into six domains: 1) surgeon experience; 2) classification of fracture types; 3) treatment options; 4) technical considerations in the operative technique; 5) predictors of patient outcome; and 6) patient outcomes. We pre-tested the 8-page questionnaire to establish its comprehensibility, face validity, and content validity [18].

### Study Sample

Of the 453 members of the Orthopaedic Trauma Association (OTA) listed on the 2002 membership list, we included all active, international, emeritus, and associate members, but excluded 11 members who are not surgeons. Therefore, we included all 442 surgeon–members of the OTA. We obtained the email addresses for the surgeons in the Internet group from the OTA's online directory.

**Figure 1:**
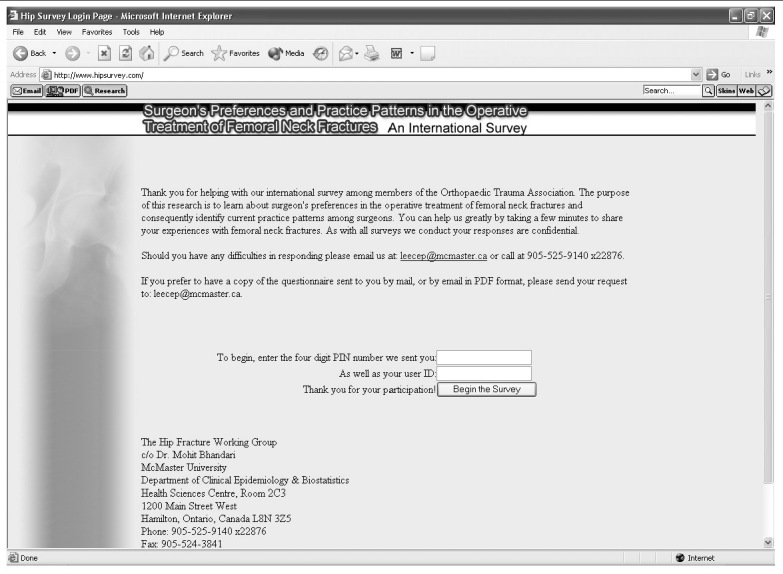
Welcome screen for Internet questionnaire

### Development of Web Questionnaire

We hired a professional Web designer to create an Internet version of the questionnaire on a Web site. The questions were displayed in the same order and format as they were in the paper version. The “welcome screen” of the Web site invited participants to enter their assigned personal identification number (PIN) and user identification (user ID) before beginning the questionnaire, so that only those in the Internet group had access to the questionnaire (Figure 1).

We also included our contact information, as well as the option to request a questionnaire by mail, fax, or email as a Portable Document Format (PDF) attachment. The Web questionnaire was 6 pages long (1 page per section), took approximately 5 minutes to complete, and had 38 questions. The responses to the Internet questionnaire were automatically entered into a database.

### Study Design and Allocation

We alternately assigned the surgeon–members of the OTA to receive a postal or an Internet questionnaire. One of us (PL), who did not know the surgeons, prepared the allocation schedule for each of the 442 surgeon–members of the OTA by using the association's membership list and, starting at the top of the alphabetical membership list, alternately assigning each name to the mail or Internet group using a systematic sampling approach. Of the 221 surgeons originally assigned to the Internet group, 45 did not have email addresses and thus received the mail version and reminders in the same way as those in the mailed questionnaire group. We selected 45 surgeons from the mail group known to have email addresses to receive the electronic questionnaire.

One of us (PL) recorded the costs associated with development and implementation of the mail and Internet-based surveys to assess the feasibility of each method. Our costs included labor, supplies, postage, Web-site administration, and our domain name. These costs were calculated and compared between groups.

### Questionnaire Administration

We planned five points of contact for the questionnaire administration: 1) advanced notification by post (mail group) or email (Internet group) 2 to 5 days prior to receiving the survey; 2) a mailed copy of the survey, or an email with a link to the Internet survey; 3) another mailed copy or email with a link to the survey at 6 weeks; 4) a further copy or link at 12 weeks; and 5) a copy of the survey sent by mail only to all non-respondents in both groups (22 weeks for the mail group and 19 weeks for the Internet group). We conducted the final mail-out to non-responders in both groups at the same time, and stopped the study for both groups at the same time, although the mail group had started three weeks before the Web site was ready for the Internet group. We calculated our primary response rates based on the number of responses received before the final mail-out; it was at that final mail-out that we changed our method of administration.

Our University Research Ethics Board reviewed and approved this research.

### Statistical Analysis

We analyzed all participants according to their final group (per protocol analysis) and the group to which they were originally assigned, following the intention to treat (ITT) principle. We summarized response rates by the proportion of respondents at each time point. Chi-square analyses were used to compare the proportion of respondents in the mail group with the proportion in the Internet group using the MINITAB version 14.0 statistical software package. All statistical tests were two-sided, at a pre-determined alpha level of 0.05.

## Results

Of the 442 surgeons, 221 received a copy of the questionnaire by mail, and 221 received an email invitation to complete the survey online. Characteristics of respondents (age, geographic location, type of practice, and the proportion who had completed a fellowship in trauma) were not different between groups (Table 1).

**Table 1:** Characteristics of the surgeons who responded to the survey before the final mailing (Intention to Treat Analysis)

Physician Characteristic		Mail (n=129)	Internet (n=99)	P-Value
Age	Under 40	25/129 (19%)	19/99 (19%)	>0.99
	41-50	64/129 (50%)	42/99 (43%)	0.31
	51-60	33/129 (26%)	25/99 (26%)	0.99
	Over 60	11/129 (9%)	6/99 (6%)	0.49
Geographic location (% North America)		104/129 (81%)	78/99 (79%)	0.73
Type of practice (% academic)		104/129 (81%)	73/99 (74%)	0.22
Trauma fellowship (% yes)		88/129 (68%)	73/99 (74%)	0.36

**Figure 2:**
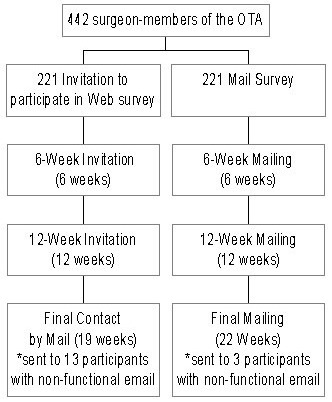
Participant flow (Intention to Treat Analysis)

**Table 2:** Geographical location of the surgeons who responded to the survey before the final mailing (Intention to Treat Analysis)

	Mail (n=129)	Internet (n=99)	Total (n=228)
Africa	1	0	1
Asia	4	3	7
Australia	0	1	1
Europe	19	16	35
North America	104	78	182
South America	0	1	1

The surgeons who responded live in 17 countries on 6 continents; 80% of all respondents before the final mailing lived in the USA (Table 2).

In the original mail group, 9 surveys were returned to sender (ie, wrong address), 3 email addresses were non-functional, and 19 surgeons explicitly refused to participate by the time we closed the study. In the original Internet group, 2 surveys were returned to sender, 13 email addresses were non-functional, and 20 people explicitly refused to participate by the end of the study (Figure 2).

There was no significant difference between the proportion of respondents who switched from the mail to the Internet group and those who switched from the Internet to the mail group (27/45 vs 22/45, P=0.287).

**Table 3:** Response rates over time (Intention to Treat Analysis)

		Mail (N=176)	Internet (N=221)	Overall (N=442)	P-Value
RESPONSES	6 weeks	75 (43%)	52 (24%)	135 (30%)	<0.01
	12 weeks	98 (56%)	77 (35%)	190 (43%)	<0.01
	22 weeks (mail) 19 weeks (Internet)	109 (62%)	99 (45%)	227 (51%)	<0.01
	32 weeks (mail) 29 weeks (Internet)	113 (64%)	141 (64%)	281 (64%)	>0.99

**Table 4:** Cost of administering survey by group

	Mail (Can $)	Internet (Can $)
Survey mailing materials	1319.41	215.78
Web administration (programming and domain name)	N/A	2413.51
Labor for mailing/ emailing ($20/hr, 5 min per mailed survey, 1 min per emailed survey)	1181.66	392.68
Labor for data entry ($20/hr, 5 min per survey)	238.33	80.00
TOTAL	2739.40	3101.95

**Figure 3:**
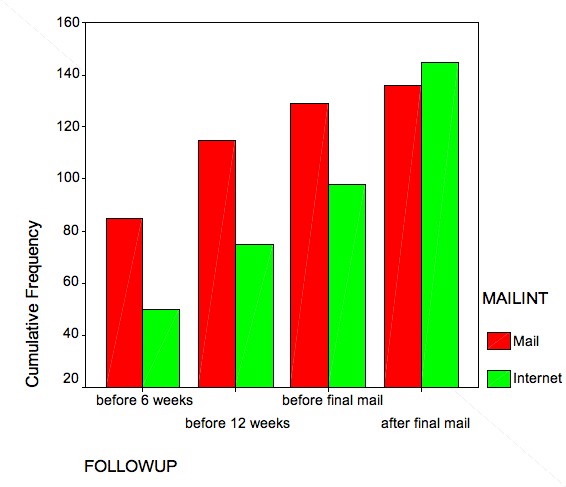
Response rates over time (per protocol analysis)

The overall primary response rate was 228/442 (51%). A significantly greater proportion of participants in the mail group responded (129/221, 58%) compared with the Internet group (99/221, 45%) (absolute difference 13%, 95% confidence interval: 4%-22%, P<0.01). The per protocol analysis similarly favored the mail group (absolute difference 14%, 95% confidence interval: 5%-23%).

The final response rate for the survey, after we had used a mixture of administration methods to raise the response rate, was 64% (281/442). Response rates did not differ significantly between the mail and Internet groups either in the intention to treat (Table 3) or per protocol analysis (Figure 3) (absolute difference: 0%, versus 4%, 95% confidence interval: -13% to 5%, respectively).

The Internet-based survey was more costly to implement than the mail survey (Can $3101.95 and Can $2739.40, respectively) (Table 4).

Had we utilized an existing Web site for developing surveys [19], the Internet costs could have been reduced to Can $968.46 for the Internet group; however, we would have been constricted in the format and design of the Web page.

## Discussion

It is important to achieve the highest response rate possible in order to limit non-response bias in health-care surveys. Previous research has demonstrated that monetary incentives, stamped return envelopes, telephone reminders, shorter surveys, and high interest can sometimes increase response rates [1,2]. Currently, there are very few data comparing response rates between postal and Internet surveys.

We hypothesized that we might receive a higher response rate among surgeons to the Web questionnaire than to the conventional paper version. We expected that surgeons with busy schedules might find the Web questionnaire would take less time and eliminate the inconvenience of dealing with paper or mailing. Additionally, we believed that widely available Internet access throughout operating suites, hospital wards, and surgeons' offices would facilitate the early completion and return of Internet-based surveys. Finally, the novelty of participating in a Web questionnaire might have interested participants who would not have completed a mailed questionnaire.

Contrary to our hypothesis, but consistent with previous studies [14,17], we found a lower response rate to the Internet questionnaire. Raziano et al randomized 2 cohorts of geriatric division chiefs to receive a survey either by electronic mail (n = 57) or by conventional postal mail (n = 57) [17]. The aggregate response rate was 58% (n = 31) for the email group versus 77% (n = 44) for the postal mail group. In another study, Kim and colleagues sent postal or email surveys to 2502 members of the American Urological Association [15]. From the postal group (n = 1000), 419 responses were obtained (42%); from the email group (n = 1502), 160 (11%) responses were obtained [15]. McMahon and colleagues compared email and postal survey response rates in a survey of physicians listed in the membership directory of the Georgia Chapter of the American Academy of Pediatrics [14]. The response rate after the first 2 mailings (2 weeks and 4 weeks) was 41% (59/143) for postal and 26% (33/125) for email surveys [14]. Harewood distributed a survey to patients about their experience after routine outpatient endoscopy. Patients were randomized to receive the questionnaire by standard mail or email. The email version of the survey resulted in a 15% lower response rate (70% vs 85%) (Table 5) [16].

**Table 5:** Response rates in previous surveys comparing mail and Internet surveys

Authors	Participants	Groups	Response
Raziano et al [17]	Geriatric division chiefs (n=114)	Email (n=57) Mail (n=57)	Email 58% (31/53)* Mail 77% (44/57)
Kim et al [15]	American Urological Association (n=2502)	Email (n=1502) Mail (n=1000)	Email 11% (160/1502) Mail 42% (419/1000)
McMahon et al [14]	Georgia Chapter of the American Academy of Pediatrics (n=268)	Email (n=125) Mail (n=143)	Email 26% (33/125) Mail 41% (59/143)
Harewood et al [16]	Patients after routine outpatient endoscopy (n=43)	Email (n=23) Mail (n=20)	Email 70% (16/23) Mail 85% (17/20)
Present study	Orthopaedic surgeon– members of the Orthopaedic Trauma Association (n=442)	Email (n=221) Mail (n=221)	Email 45% (99/221) Mail 58% (128/221)* after final mailing to all: Email 64% (141/221) Mail 63% (140/221)

^*^ 4 individuals had incorrect or no email address

This is also consistent with Couper's caution that for using a probability-based method, with a list-based sample of high-coverage populations, non-response remains a concern. People will usually choose a paper version over an Internet version of a survey [12].

We found a lower response rate to the Internet questionnaire despite efforts to make the Internet version of our questionnaire easy to use, and despite the inclusion of a link to the Web site in the invitation email. We followed closely the recommendations for conducting Web surveys made by Dillman, who reported comparable electronic and postal mail response rates [9]. We have also avoided many of the common problems with Internet surveys noted by Zhang: our design used a population that has easy access to the Internet and that is relatively comfortable with it; we eliminated self-selection bias and increased the validity of responses by using ID; we used a personalized survey; and we blocked participants from entering multiple responses [20].

However, we were probably able to achieve similar final response rates for those who originally received the survey by Internet only because we used mixed modes (ie, sent by email, offered PDF, and finally sent a paper copy by mail), as shown by our response rates up until the final reminders (Table 3).

There are several possible explanations for why the response rate was lower for the Web questionnaire. It may be that participants tend to be worried about computer viruses and delete emails that are unsolicited or from someone they do not know. In fact, it may be easier to delete an email than it is to ignore a mailed survey. It may also be that more paper surveys sent to the incorrect address may have been forwarded to participants, whereas emails would not be re-directed (however, we did not find a significant difference in the number of returned emails versus paper surveys). Having to enter a user ID and PIN to access the Internet questionnaire may have deterred participants. Several participants who used Netscape as their browser contacted us to report that they had trouble navigating through the pages of the survey. We expected that the level of computer literacy in this group would be quite high, although this may not have been the case. The use of different versions or types of browsers and different operating platforms can result in the questionnaire being displayed differently on the designer's computer and the respondent's computer [9]. Other differences in the respondent's computer equipment can affect the appearance of the questionnaire or the ease of using it. Differences include the configuration of the user's screen resolution, Internet connection speed, memory resources, and software applications [9].

In the end, the cost of using the Web site was higher than mailing the survey (Can $3101.95 vs Can $2739.40) because of the cost of Web programming and the monthly cost of the domain name. Our decision to design a custom Web page for the survey led to the increased cost of the Internet survey. Had our sample size been larger, the cost of the Web survey would have been less than the cost of the mailed survey: set-up costs for the Web survey were high, but the cost per additional participant was low [21].

In retrospect, excluding those without email addresses and randomizing the remainder represents a superior design to the one we chose, which requires separate consideration of per protocol and intention-to-treat analyses. However, results were very similar in the two analyses. Our allocation method was “pseudo-random” because we did not use a random number generator to allocate participants to each group. However, our method probably produced the same effect as randomization because we alternately assigned participants to groups using an alphabetical list. Therefore, the assignment of participants was not based on any factor that could plausibly affect their inclination to respond.

We also did not pre-determine whether participants were regular Internet users, or ask non-responders why they did not complete our questionnaire. Thus, it remains possible that more selective use of Internet users would lead to higher response rates. We do not feel that the email group's receiving the final mail-out three weeks later than the mail survey group had much effect on the response rates. Because email communication is much faster than postal mail, we found that after each reminder, responses from the Internet group stopped coming in much earlier than those from the postal mail group. Although one might also challenge the generalizability of our results to surgeons beyond the membership of the OTA, the similar findings of other studies suggest the results may be broadly generalizable. Another limitation of this study is that we cannot precisely measure the reception of the survey by mail and Internet: if the reception differs by the mode, the response rate could be confounded if those who did not receive the survey were included in the denominator. To be conservative we have included in the denominator all those we tried to reach.

We conclude that postal surveys still result in higher initial response rates than Internet-based surveys. Researchers should not assume that the widespread availability and potential ease of Internet-based surveys will translate into higher response rates. Future research should focus on how to refine our techniques in conducting Internet surveys so that they are more accessible and easier to use. Asking non-respondents to Internet-based surveys why they did not respond will inform this work. As our expertise increases in the area of conducting Internet surveys, we will be able to make a more informed evaluation of whether they constitute a valuable tool for conducting health research.
