# Correction and Republication: Internet Versus Mailed Questionnaires: A Controlled [Correction of "Randomized"] Comparison (2)

**DOI:** 10.2196/jmir.6.4.e38

**Published:** 2004-10-29

**Authors:** Pam Leece

A number of errors occurred in the article by Pam Leece et al. (J Med Internet Res 2004;6(3):e30). The corrected version has been republished as J Med Internet Res 2004;6(4):e39 and is available at http://www.jmir.org/2004/3/e39/. Please do not cite the old version.

The title of the article should have been "Internet Versus Mailed Questionnaires: A Controlled Comparison" (rather than "Internet Versus Mailed Questionnaires: A Randomized Comparison"), because the subjects were alternately assigned to receive a postal or an Internet questionnaire, thus the allocation process was not truly random, but pseudo-random. The authors have also noted an error in their previous analysis and changed the number of primary responders in mail group to 128, rather than 129. This change also affects Table 1 and Table 2, so that the denominator in the mail group should be 128 instead of 129 and the total respondents to be 227 instead of 228. Some of the numerators in this table were also revised after the authors noted an additional error in which respondents were attributed to each group. The original Figure 2 that was published omitted information at the bottom of the figure that we intended to be included, so this figure has also been updated. Due to a revised intention to treat analysis of the results to include all participants we contacted in both groups, Table 3 has been revised to include 221 participants in the denominator rather than 176 as originally published. Finally, the original publication included a figure (Figure 3) that represented the cumulative frequency of responses by group and by follow-up contact according to a per protocol analysis. This figure has been removed to avoid confusion and to keep the intention to treat analysis as the primary analysis of the results consistent throughout the paper.

